# Four Antioxidant Peptides from Protein Hydrolysate of Red Stingray (*Dasyatis akajei*) Cartilages: Isolation, Identification, and In Vitro Activity Evaluation

**DOI:** 10.3390/md17050263

**Published:** 2019-05-03

**Authors:** Xiao-Yang Pan, Yu-Mei Wang, Li Li, Chang-Feng Chi, Bin Wang

**Affiliations:** 1 National and Provincial Joint Laboratory of Exploration and Utilization of Marine Aquatic Genetic Resources, National Engineering Research Center of Marine Facilities Aquaculture, School of Marine Science and Technology, Zhejiang Ocean University, Zhoushan 316022, China; 18368091610@163.com (X.-Y.P.); wenwenlili@163.com (L.L.); 2 Zhejiang Provincial Engineering Technology Research Center of Marine Biomedical Products, School of Food and Pharmacy, Zhejiang Ocean University, Zhoushan 316022, China; wangym731@126.com

**Keywords:** red stingray (*Dasyatis akajei*), cartilage, peptide, antioxidant activity

## Abstract

In the work, water-soluble proteins of red stingray (*Dasyatis akajei*) cartilages were extracted by guanidine hydrochloride and hydrolyzed using trypsin. Subsequently, four antioxidant peptides (RSHP-A, RSHP-B, RSHP-C, and RSHP-D) were isolated from the water-soluble protein hydrolysate while using ultrafiltration and chromatographic techniques, and the amino acid sequences of RSHP-A, RSHP-B, RSHP-C, and RSHP-D were identified as Val-Pro-Arg (VPR), Ile-Glu-Pro-His (IEPH), Leu-Glu-Glu--Glu-Glu (LEEEE), and Ile-Glu-Glu-Glu-Gln (IEEEQ), with molecular weights of 370.46 Da, 494.55 Da, 647.64 Da, and 646.66 Da, respectively. VPR, IEPH, LEEEE, and IEEEQ exhibited good scavenging activities on the DPPH radical (EC_50_ values of 4.61, 1.90, 3.69, and 4.01 mg/mL, respectively), hydroxyl radical (EC_50_ values of 0.77, 0.46, 0.70, and 1.30 mg/mL, respectively), superoxide anion radical (EC_50_ values of 0.08, 0.17, 0.15, and 0.16 mg/mL, respectively), and ABTS cation radical (EC_50_ values of 0.15, 0.11, 0.19, and 0.18 mg/mL, respectively). Among the four isolated antioxidant peptides, IEPH showed the strongest reducing power and lipid peroxidation inhibition activity, but LEEEE showed the highest Fe^2+^-chelating ability. The present results suggested that VPR, IEPH, LEEEE, and IEEEQ might have the possibility of being an antioxidant additive that is used in functional food and pharmaceuticals.

## 1. Introduction

Superfluous reactive oxygen species (ROS) are produced under oxidative stress conditions and they can destroy all types of macromolecules, which further lead to many health disorders, such as cancer, diabetes mellitus, and inflammatory diseases [1,2,3]. In addition, oxidative decomposition of unsaturated lipids causes food rancidity and shortens the shelf-life of food products [4,5,6,7]. Antioxidant peptides (APs) from food resources usually contain 2–20 amino acid residues and they can donate hydrogen, trap lipid-derived free radicals, and/or activate antioxidant enzymes in cells [5,6,7,8,9,10]. Therefore, APs can convert ROS, including superoxide anion radicals ($O_{2}^{-}$•) and hydroxyl radicals (HO•) into stable molecular structure to inhibit the propagation of peroxidizing chain reaction [5,6,7,8,9,10]. FWKVV and FMPLH were isolated from the protein hydrolysate of miiuy croaker (*Miichthys miiuy*) muscle and exhibited strong reducing power, lipid peroxidation inhibition ability, and radical scavenging activity [9]. The AP fractions of CPF1 (molecular weight (MW) < 1 kDa) and CPF2 (1 < MW < 3 kDa) from corn gluten exhibited cytoprotective effect and intracellular ROS scavenging activity in H_2_O_2__-_damaged HepG2 cells because they could positively affect the activities of the intracellular antioxidant enzymes [11]. These studies suggested that APs could effectively enhance human health and/or protect food quality by reducing the oxidative stress [12]. In addition, APs can serve as ingredients of functional foods due to their nutritional and biological properties [1,13].

Collagens and peptides from porcine and cow cartilages can provide an adequate nutrient for repairing cartilage, maintain the overall health of subchondral bone and articular cartilage, and improve the joint flexibility and mobility in Osteoarthritis (OA) patients [14,15]. However, these ingredients that were used in food and medical products have caused anxiety among some consumers due to frequent outbreaks of infectious disease [15]. Therefore, cartilages from Chondrichthyes, such as sharks, skates, and rays, are thought to be one of the alternatives of porcine and cow cartilages, because they contain analogous bioactive substances, such as active proteins (angiogenesis inhibitors), proteoglycan, collagen, bioactive peptides, and elastin fibers [16,17,18,19,20,21,22,23]. Furthermore, peptides from protein and collagen hydrolysates of fish cartilages showed a variety of biological activities [15,18,19]. The polypeptide (PG155) with molecular weight (MW) of 15.500 kDa from the cartilages of *Prionace glauca* can significantly reduce the growth of subintestinal vessels (SIVs) in the zebrafish embryos model. PG155 also can inhibit vascular endothelial growth factor (VEGF) induced the migration and tubulogenesis of HUVECs [17]. FIMGPY, IVAGPQ, and IVAGPQ from *Raja porosa* cartilages exhibited high lipid peroxidation inhibiting power and radical scavenging activities [20,21]. In addition, FIMGPY could induce the apoptosis of HeLa cells by upregulating the Bax/Bcl-2 ratio and activating caspase-3 [21]. GAERP, GEREANVM, and AEVG from protein hydrolysate of *Sphyrna lewini* cartilages exhibited good radical scavenging activities, similar inhibiting ability on lipid peroxidation with butylated hydroxytoluene (BHT), and protection on H_2_O_2_-damaged HepG2 cells by decreasing the content of malonaldehyde (MDA) and increasing the levels of antioxidant enzymes [22]. The cartilage collagens and peptides showing significant pharmacological effects on repairing cartilage and maintaining the overall health of subchondral bones and articular cartilage collagens is more noteworthy [15,19,23]. 

Red stingray (*Dasyatis akajei*), which mainly resides in the northwestern Pacific Ocean, is a common species of stingray in the Dasyatidae family. In previous studies, some active substances, including proteins (angiogenesis inhibitors), polysaccharides, collagens, and collagen hydrolysates, were prepared from its cartilages [16,24,25]. Luo et al. reported that an angiogenesis inhibitor (DCAI-1) with a MW of 62 kDa from red stingray cartilages could inhibit the formation of the blood vessels of the chick embryo chorioallantoic membrane in a dose-dependent manner [24,26]. The mucopolysaccharides from red stingray cartilages could significantly reduce the values of total cholesterol, triglyceride, and low-density lipoprotein of blood serum, but increase the value of high-density lipoprotein cholesterol in hyperlipidaemia rabbits [27]. Chi et al. isolated and characterized the acid-soluble collagen (ASC) from red stingray cartilages, and the results indicated that the ASC was comprised of type I and II collagens. In addition, the properties of cartilage collagen hydrolysates from *D. akjei*, *S. lewini*, and *R.* *porosa* indicated that the antioxidant activities of collagen hydrolysates were negatively correlated with the logarithm of their average MWs [25]. However, there is no report on the APs from red stingray cartilages. Therefore, in the present study, peptides from water-soluble protein hydrolysates of red stingray cartilages were purified and characterized, and their *in vitro* antioxidant activities were then evaluated.

## 2. Results and Discussion

### 2.1. Isolation of APs from Water-Soluble Protein Hydrolysate (RSH) of Red Stingray Cartilages

#### 2.1.1. Fractionation of RSH by Ultrafiltration

Bioactive peptides are inactive in the sequence of their parent proteins and they can be released by enzymatic hydrolysis either during gastrointestinal digestion in the body or during food processing [2]. Bioactive peptides may act as regulatory compounds with diverse biological activities once they are liberated from protein sources by proteolysis, such as antioxidant, antihypertensive, and enhancing immunity activities [1]. Subsequently, water-soluble proteins of red stingray cartilages were hydrolyzed using trypsin in the experiment.

**Figure 1:**
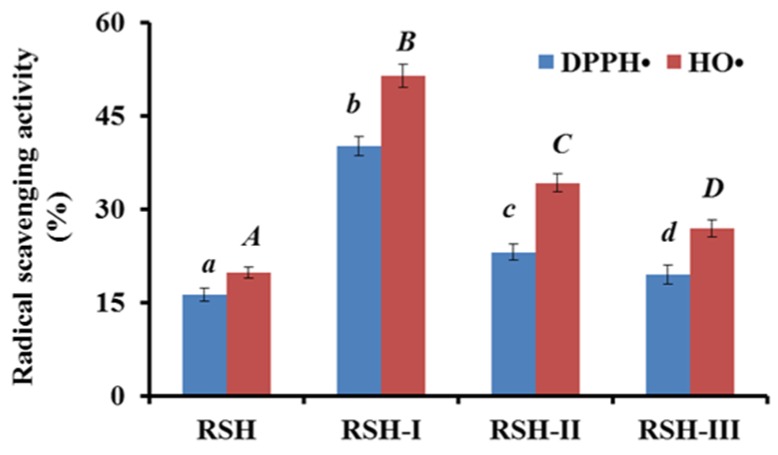
2,2-diphenyl-1-picrylhydrazyl radical (DPPH•) and hydroxyl radical (HO•) scavenging activity of protein hydrolysate (RSH) and its three fractions by ultrafiltration at the concentration of 10 mg protein/mL. All data were presented as mean ± standard deviation (SD, *n* = 3). a–d or A–D, Values with same letters indicated no significant difference of different samples on DPPH• or HO• scavenging activity (*p* > 0.05).

Three fractions RSH-I (MW < 3 kDa), RSH-II (3 < MW < 10 kDa), and RSH-III (MW > 10 kDa) were prepared from protein hydrolysate (RSH) of red stingray cartilages using 3 and 10 kDa ultrafiltration membranes, and their radical scavenging activities are shown in Figure 1. The 2,2-diphenyl-1-picrylhydrazyl radical (DPPH•) and HO• scavenging rates of RSH-I were 40.12 ± 1.57% and 51.43 ± 1.88% at the concentration of 10 mg protein/mL, which were significantly (*p* < 0.05) higher than those of RSH, RSH-II, and RSH-III at the same concentration. Short chain peptides easily access free radicals and act as electron donors to convert them into the stable state and inhibit the chain reactions [25,28]. The results were in agreement with previous reports that fractions from protein hydrolysates with low molecular size had high antioxidant activities, such as fractions from protein hydrolysates of miiuy croaker muscle [9], cod muscle [29], skate cartilage [21], bluefin leatherjacket head [30], and blue mussel [2,31]. Therefore, RSH-I was chosen for the preparation of APs with high activity.

#### 2.1.2. Anion-Exchange Chromatography of RSH-I

**Figure 2:**
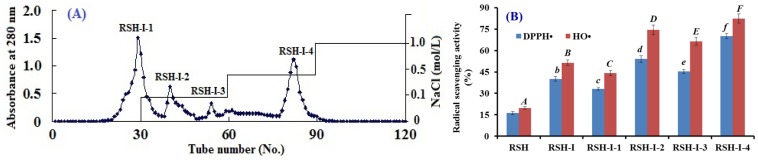
Elution profile of RSH-I through DEAE-52 cellulose anion-exchange chromatography (**A**) and DPPH• and HO• scavenging activities of RSH-I and its fractions at the concentration of 10 mg protein/mL (**B**). All data were presented as mean ± SD (*n* = 3). a–f or A–F, Values with same letters indicated no significant difference of different samples on DPPH• and HO• scavenging activity (*p* > 0.05).

Figure 2A showed that four fractions (RSH-I-1 to RSH-I-4) were separated from RSH-I using a DEAE-52 cellulose column. Among them, RSH-I-1 was eluted using deionized water (DW); RSH-I-2 and RSH-I-3 were eluted using 0.1 M NaCl; and, RSH-I-4 was eluted using 0.5 M NaCl. Figure 2B indicated that the DPPH• and HO• scavenging activities of RSH-I-4 were 70.2 ± 1.8% and 82.4 ± 3.20% at the concentration of 10 mg protein/mL, which were significantly (*p* < 0.05) higher than those of RSH-I, RSH-I-1, RSH-I-2, and RSH-I-3 at the same concentration. Acidic and hydrophobic amino acid residues in peptides can be easily adsorbed to the anion exchange resins, and the interaction strength is closely related to the number and location of the charges on the molecule structure [25,30]. The results indicated that RSH-I-4 might contain some APs, with acidic and hydrophobic amino acid residues.

#### 2.1.3. Gel filtration Chromatography of RSH-I-4

**Figure 3:**
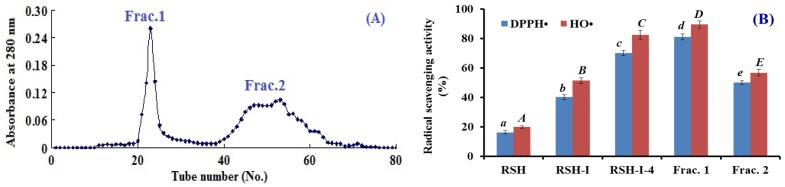
Elution profile of RSH-I-4 using Sephadex G-15 column (**A**) and DPPH• and OH• scavenging activities of subfractions (Frac.1 and Frac.2) from RSH-I-4 at the concentration of 10 mg protein/mL (**B**). All data were presented as mean ± SD (*n* = 3). a–e or A–E, Values with same letters indicated no significant difference of different samples on DPPH• and HO• scavenging activity (*p* > 0.05).

As shown in Figure 3A, RSH-I-4 was subsequently separated into two subfractions (Frac.1 and Frac.2) by a Sephadex G-15 column. The radicals scavenging rates of Frac.1 on DPPH• and OH• were 81.1 ± 1.98% and 89.5 ± 2.37% at the concentration of 10 mg protein/mL, which were significantly (*p* < 0.05) higher than those of RSH-I-4 and Frac.2 (Figure 3B). Smaller peptide fractions generally exhibited a stronger antioxidant activity than their larger counterparts [32,33]. However, Frac.1 with bigger MW showed a higher activity than Frac.2 did, which indicated that some other factors, such as amino acid residue composition and spatial conformation, also played important roles in the bioactivity of APs.

#### 2.1.4. Isolation of APs from Frac.1 by RP-HPLC

**Figure 4:**
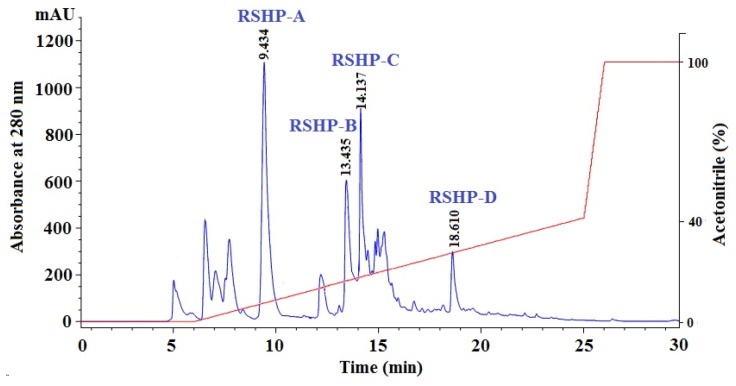
Elution profile of Frac.1 separated by RP-HPLC on a Thermo C-18 column (4.6 × 250 mm) from 0 to 30 min.

Finally, Frac.1 with high DPPH• and HO• scavenging activities was purified by RP-HPLC while using a linear gradient of acetonitrile (CAN) (Figure 4). All of the chromatographic peaks were collected on their chromatographic peaks and their purities were analyzed. Finally, four APs (RSHP-A, RSHP-B, RSHP-C, and RSHP-D) with retention times of 9.434, 13.435, 14.137, and 18.610 min. had high purities and met the requirement of sequence determination (Table 1). Therefore, RSHP-A, RSHP-B, RSHP-C, and RSHP-D were pooled and lyophilized, and their sequences and activities were determined in the following experiment.

### 2.2. Amino Acid Sequence and Mass Spectrometry Analysis of APs (RSHP-A~D)

**Figure 5:**
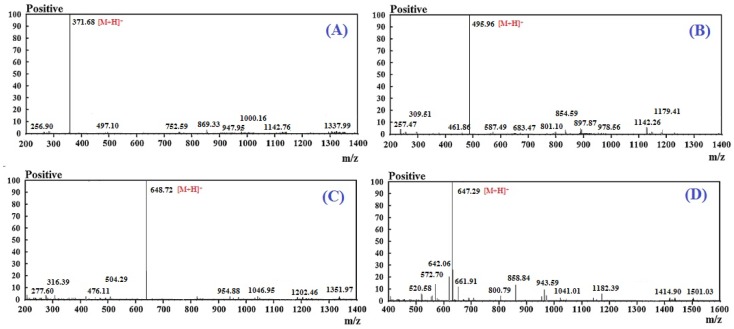
Mass spectrogram of RSHP-A (**A**), RSHP-B (**B**), RSHP-C (**C**), and RSHP-D (**D**).

Using a Protein/Peptide Sequencer and electrospray ionization mass spectrometer (ESI-MS), the amino acid sequences of RSHP-A, RSHP-B, RSHP-C, and RSHP-D were determined to be Val-Pro-Arg (VPR), Ile-Glu-Pro-His (IEPH), Leu-Glu-Glu--Glu-Glu (LEEEE), and Ile-Glu-Glu-Glu-Gln (IEEEQ), respectively. The measured MWs of RSHP-A, RSHP-B, RSHP-C, and RSHP-D were 370.46 Da ([M + H]^+^ 371.68 Da), 494.55 Da ([M + H]^+^ 495.96 Da), 647.64 Da ([M + H]^+^ 648.72 Da), and 646.66 Da ([M + H]^+^ 647.29 Da) (Figure 5 and Table 1), which agreed well with their theoretical masses of 370.44 Da, 494.52 Da, 647.69 Da, and 646.62 Da, respectively.

### 2.3. Antioxidant Activity

#### 2.3.1. Radical Scavenging Activity

##### DPPH• Scavenging Activity

**Figure 6:**
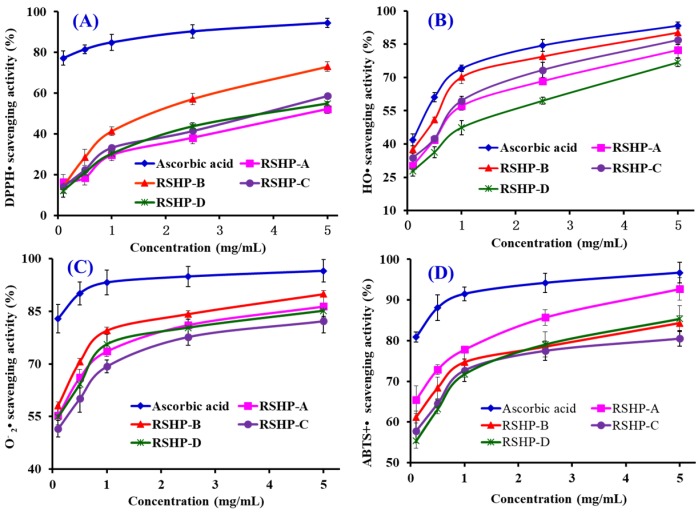
DPPH• (**A**), HO• (**B**), $O_{2}^{-}$• (**C**), and ABTS^+^• (**D**) scavenging activities of RSHP-A, RSHP-B, RSHP-C, and RSHP-D from protein hydrolysate of red stingray (*D. akajei*) cartilages. All data are presented as mean ± SD (*n* = 3).

The activity curves on DPPH• scavenging of four isolated APs (RSHP-A~D) are shown in Figure 6A, and a positive correlation between the APs concentrations and their radical-scavenging activities was observed. The EC_50_ value of RSHP-B was 1.90 mg/mL, which was significantly (*p* < 0.05) higher than those of RSHP-A (4.61 mg/mL), RSHP-C (3.69 mg/mL), and RSHP-D (4.01 mg/mL), respectively (Table 1). Furthermore, the EC_50_ value of RSHP-B was lower than those of the APs from protein hydrolysates of croceine croaker muscle (YLMSR: 2.74 mg/mL; MILMR: 5.01 mg/mL) [34], loach (PSYV: 17.0 mg/mL) [35], blue mussel (TTANIEDRR: 2.50 mg/mL) [31], salmon (FLNEFLHV: 4.95 mg/mL) [3], bluefin leatherjacket head (WEGPK: 4.44 mg/mL; GVPLT: 4.54 mg/mL) [30], grass carp skin (GFGPL: 2.25 mg/mL; VGGRP: 2.94 mg/mL) [36], skate cartilage (FIMGPY: 2.60 mg/mL; GPAGDY: 3.48 mg/mL; IVAGPQ: 3.93 mg/mL) [21], and scalloped hammerhead muscle (WDR: 3.63 mg/mL; PYFNK: 4.11 mg/mL; LDK: 3.06 mg/mL) [37,38]. However, the EC_50_ value of RSHP-B was higher than those of APs from protein hydrolysates of miiuy croaker swim bladder (FPYLRH: 0.51 mg/mL; GIEWA: 0.78 mg/mL) [39], blue mussel (PIIVYWK: 0.71 mg/mL; FSVVPSPK: 0.94 mg/mL) [31], skipjack tuna bone (GADIVA: 0.57 mg/mL; GAEGFIF: 0.30 mg/mL) [40], grass carp skin (PYSFK: 1.58 mg/mL) [36], and bluefin leatherjacket skin (GSGGL: 0.41 mg/mL; GPGGFI: 0.19 mg/mL; FIGP: 0.12 mg/mL) [41]. The radical-quenching activities of APs are due to their ability to participate in the single electron transfer reaction [1]. The present results illustrated that RSHP-B have a strong ability to act as a free radical scavenger or hydrogen donor to transform DPPH• into harmless compounds and to prevent the single electron transfer reaction.

##### HO• Scavenging Activity

The HO• scavenging activity of four isolated APs (RSHP-A~D) was dose-dependent at the test concentrations (Figure 6B). RSHP-B exhibited the highest HO• scavenging activity, with an EC_50_ value of 0.45 mg/mL, which was significantly (*p* < 0.05) stronger than those of RSHP-A (0.76 mg/mL), RSHP-C (0.70 mg/mL), and RSHP-D (1.30 mg/mL), respectively (Table 1). The EC_50_ value of RSHP-B was lower than those of APs from the protein hydrolysates of conger eel (LGLNGDDVN: 0.69 mg/mL) [42], weatherfish loach (PSYV: 2.64 mg/mL) [35], miiuy croaker muscle (FWKVV: 0.97 mg/mL; FMPLH: 0.80 mg/mL) [9], mussel sauce (HFGDPFH: 0.50 mg/mL) [43], blue mussel (YPPAK 0.23 mg/mL) [2], hairtail muscle (KA: 1.74 mg/mL; AKG: 2.38 mg/mL; IYG: 2.50 mg/mL) [44], grass carp (PSKYEPFV: 2.86 mg/mL; PYSFK: 2.28 mg/mL; GFGPL: 1.61 mg/mL; VGGRP: 2.06 mg/mL) [36,45], miiuy croaker swim bladder (FPYLRH: 0.68 mg/mL; GIEWA: 0.71 mg/mL) [39], giant squid (NADFGLNGLEGLA: 0.61 mg/mL) [43], and skate cartilage (FIMGPY: 3.04 mg/mL; GPAGDY: 3.92 mg/mL; IVAGPQ: 5.03 mg/mL) [21]. However, the EC_50_ value of RSHP-B was higher than those of the APs from protein hydrolysates of scalloped hammerhead muscle (PYFNK: 0.24 mg/m; LDK: 0.17 mg/mL) [37,38], skipjack tuna bone (GADIVA: 0.25 mg/mL; GAEGFIF: 0.32 mg/mL) [40], blue mussel (YPPAK: 0.23 mg/mL) [3], bluefin leatherjacket skin (GSGGL: 0.18 mg/mL; GPGGFI: 0.09 mg/mL; FIGP: 0.07 mg/mL) [41], and giant squid (NGLEGLK: 0.31 mg/mL) [43]. HO• is one of the most deleterious intracellular ROS and it can cause severe damage to biomacromolecules, including carbohydrates, nucleic acids (mutations), lipids (lipid peroxidation), and amino acids (e.g., conversion of Phe to m-Tyrosine and o-Tyrosine), which further causes a series of chronic diseases [46]. In addition, HO• cannot be eliminated by an enzymatic reaction, and scavenging it from organisms using antioxidants, such as glutathione, sulforaphane, a-tocopherol, curcumin, and ascorbic acid is the only mean to protect important cellular structures from its damage [22,47]. Therefore, four isolated APs, especially RSHP-B, showed a high scavenging ability on HO• and it might have the possibility to serve as a scavenger to weaken the HO• damage in biological systems.

##### $O_{2}^{-}$• Scavenging Activity

The $O_{2}^{-}$• scavenging activities of four isolated APs (RSHP-A~D) were studied, and the dose-effect relations were observed when their concentrations gradually increased from 0.1 to 5.0 mg/mL (Figure 6C). The EC_50_ values of RSHP-A, RSHP-B, RSHP-C, and RSHP-D were 0.08, 0.17, 0.15, and 0.16 mg/mL, respectively (Table 1). RSHP-A showed stronger $O_{2}^{-}$• scavenging activity than RSHP-B, RSHP-C, and RSHP-D did. The EC_50_ value of RSHP-A was lower than those of APs from protein hydrolysates of skipjack tuna bone (GADIVA: 0.52 mg/mL; GAEGFIF: 0.48 mg/mL) [40], miiuy croaker swim bladder (FPYLRH: 0.34 mg/mL; GIEWA: 0.30 mg/mL) [39], hairtail muscle (KA: 2.08 mg/mL; AKG: 2.54 mg/mL; IYG: 1.36 mg/mL) [44], mussel sauce (HFGDPFH: 0.20 mg/mL) [43], *Mytilus coruscus* (SLPIGLMIAM: 0.32 mg/mL) [48], round scad (HDHPVC: 0.27 mg/mL; HEKVC: 0.24 mg/mL) [49], miiuy croaker muscle (FWKVV: 0.29 mg/mL; FMPLH: 0.15 mg/mL) [9], croceine croaker muscle (YLMR: 0.45 mg/mL; VLYEE: 0.69 mg/mL; MILMR: 0.99 mg/mL) [34,50], and skate cartilage (FIMGPY: 1.61 mg/mL; GPAGDY: 1.66 mg/mL; IVAGPQ: 1.82 mg/mL) [21]. However, the EC_50_ value of RSHP-A was higher than those of the APs from protein hydrolysates of blue mussel (YPPAK: 0.072 mg/mL) [3] and monkfish muscle (LMGQW: 0.042 mg/mL) [51]. $O_{2}^{-}$• is the most common free radical that is generated *in vivo*, and it can promote oxidative reaction to generate hydrogen peroxide and hydroxyl radical [22]. $O_{2}^{-}$• and its derivative radicals seriously threaten the bioactive biomacromolecules and cause different diseases of vital organs of the human body [1,30]. For example, $O_{2}^{-}$• plays crucial roles in cardiovascular and neurodegenerative diseases, as well as in cancer [47]. In organism, SOD can timely catalyze $O_{2}^{-}$• into uninjurious hydrogen peroxide and oxygen under normal conditions. Subsequently, RSHP-A, RSHP-B, RSHP-C, and RSHP-D have high $O_{2}^{-}$• scavenging activity and might be applied to eliminate the $O_{2}^{-}$• damage together with SOD in biological systems. 

##### ABTS^+^• Scavenging Activity

The abilities of four isolated APs (RSHP-A~D) to scavenge ABTS^+^• in comparison with ascorbic acid were investigated, and dose-related effects were observed at peptide concentrations that ranged from 0.1 to 5.0 mg/mL (Figure 6D). The EC_50_ values of RSHP-A, RSHP-B, RSHP-C, and RSHP-D were 0.15, 0.11, 0.19, and 0.18 mg/mL, respectively (Table 1). RSHP-B showed the strongest scavenging activity among four isolated APs at all tested concentrations. The EC_50_ value of RSHP-B was lower than those of APs from the protein hydrolysates of croceine croaker muscle (VLYEE: 0.31 mg/mL; YLMSR: 0.42 mg/mL; MILMR: 0.89 mg/mL) [34], hairtail muscle (KA: 1.65 mg/mL; AKG: 0.83 mg/mL; IYG: 0.59 mg/mL) [44], scalloped hammerhead muscle (WDR: 0.34 mg/mL; LDK (0.19 mg/mL) [37,38], salmon (FLNEFLHV: 1.55 mg/mL) [3], grass carp skin (GFGPL: 0.33 mg/mL; VGGRP: 0.47 mg/mL) [36], skipjack tuna bone (GADIVA: 0.41 mg/mL; GAEGFIF: 0.21 mg/mL) [40], bluefin leatherjacket head (WEGPK: 5.41 mg/mL; GPP: 2.47 mg/mL; GVPLT: 3.12 mg/mL) [30], and skate cartilage (FIMGPY: 1.04 mg/mL; GPAGDY: 0.77 mg/mL; IVAGPQ: 1.29 mg/mL) [20]. ABTS^+^• scavenging assay has been successfully used to evaluate the capacity of dietary antioxidant ingredients, such as polyphenols, polysaccharides, and peptides based on the special chemical properties of formed free radicals [22,38]. In the assay, blue ABTS^+^• with an absorption maximum of 734 nm is inactivated to its colorless neutral form, when it meets an antioxidant compound. The present results indicated that RSHP-A, RSHP-B, RSHP-C, and RSHP-D could act as electrons or hydrogen atoms donator to inactivate ABTS^+^•.

#### 2.3.2. Fe^2+^-Chelating Ability

**Figure 7:**
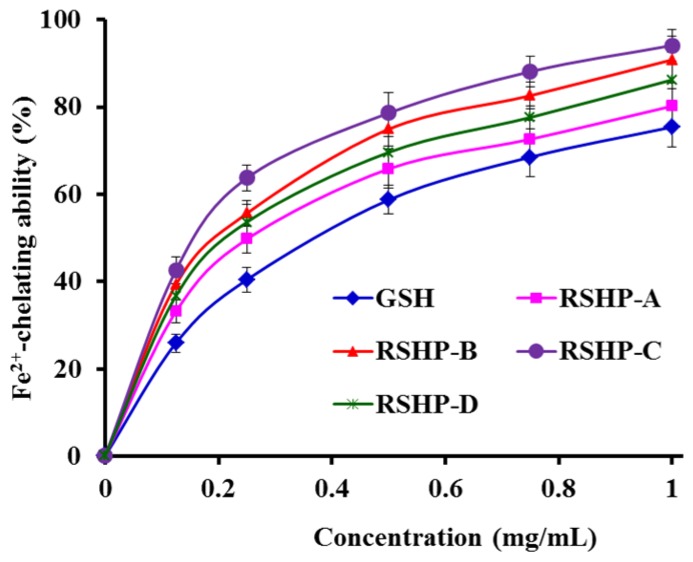
Fe^2+^-chelating ability of the four isolated APs (RSHP-A~D) from protein hydrolysate of red stingray (*D. akajei*) cartilages. All data are presented as mean ± SD (*n* = 3).

Figure 7 showed that four isolated APs (RSHP-A~D) chelated Fe^2+^ in a dose-effect manner at concentrations that ranged from 0 to 1.0 mg/mL, and RSHP-C showed the highest chelating ability on Fe^2+^ among four isolated APs (RSHP-A~D). Transition metals, such as Fe^2+^ and Cu^2+^, can serve as catalysts that accelerate the generation of ROS, which further oxidize unsaturated lipids and initiate the oxidative chain reaction [52]. In recent years, some metal chelating peptides have been identified from different food protein resources, such as oyster, anchovy, whey, soybean, and mungbean [53]. Lapsongphon and Yongsawatdigul reported that side chains, including carboxyl (Glu, Asp) and amino groups (Lys, His, Arg), can serve as metal chelators to reduce the available amount of transition metals, causing the inhibition of radical-mediated oxidative chain reactions [42]. Cruz-Huerta et al. have reported that Asp, Glu, and Pro were the most abundant amino acids in the iron-binding peptides from whey protein [54]. GPAGPHGPPGKDGR, AGPHGPPGKDGR, and AGPAGPAGAR from gelatin tryptic hydrolysates of pacific cod skin exhibit high affinity to ferrous ions. Among the groups of the three peptides, the amino and carboxylate terminal groups and peptide bond from peptide backbone, as well as the amino and imine from Arg side chain, are involved in the complexation. In addition, several amino acid side chain groups of GPAGPHGPPGKDGR and AGPHGPPGKDGR, including amino (Lys), imine (His), and carboxylate (Asp), supplied additional iron binding sites [53]. The four isolated APs (RSHP-A~D), especially RSHP-B~D, are rich in acid and basic amino acids. Therefore, they showed higher Fe^2+^-chelating ability than the positive control of GSH.

#### 2.3.3. Reducing Power

**Figure 8:**
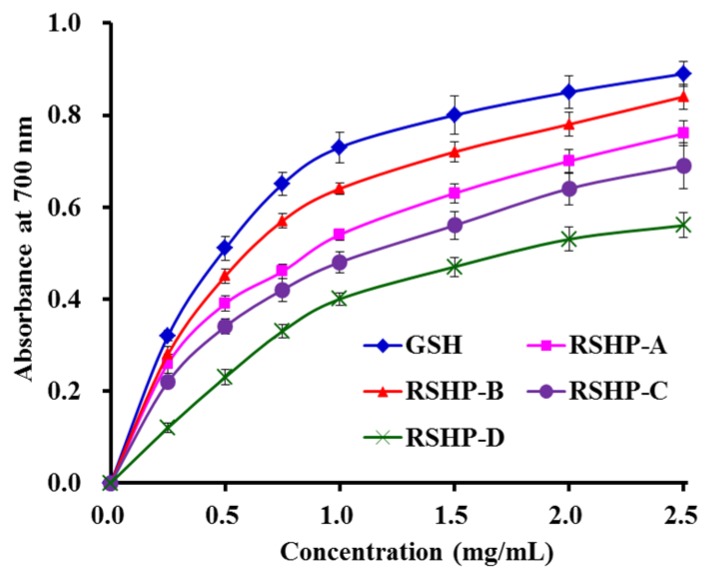
Reducing power of the four isolated APs (RSHP-A~D) from protein hydrolysate of red stingray (*D. akajei*) cartilages. All data are presented as mean ± SD (*n* = 3).

As shown in Figure 8, four isolated APs (RSHP-A~D) exhibited dose-dependent reducing power at the tested concentrations, and RSHP-B showed the highest capacity to reduce Fe^3+^ to Fe^2+^ among the four isolated APs (RSHP-A~D). In organisms, the antioxidants act as reducing agents to be oxidized for inhibiting the ongoing oxidation reactions. Subsequently, the reducing power is an important indicator to provide information on the potential of samples to serve as antioxidant agents [9,55]. 

Yang et al. reported that QNDER, KS, KA, AKG, TKA, VK, MK, and IYG from protein hydrolysate of hairtail muscle showed high reducing power, and the absorbance at 700 nm of these eight antioxidant peptides was less than 0.7 at the concentration of 2.5 mg/mL [44]. The data indicated that the reducing power of RSHP-B was stronger than those of the APs from the protein hydrolysate of hairtail muscle at the same concentration. He et al. reported that the absorbance of pentapeptide (FMPLH) from the protein hydrolysate of miiuy croaker muscle at 700 nm was approximate 0.9 at the concentration of 2.5 mg/mL, which was slightly higher than those of RSHP-A~D [9]. The present data indicated that RSHP-B might be applied as a reducing power agent to terminate the *in vivo* oxidation reactions that are in progress.

#### 2.3.4. Lipid Peroxidation Inhibition Ability

**Figure 9:**
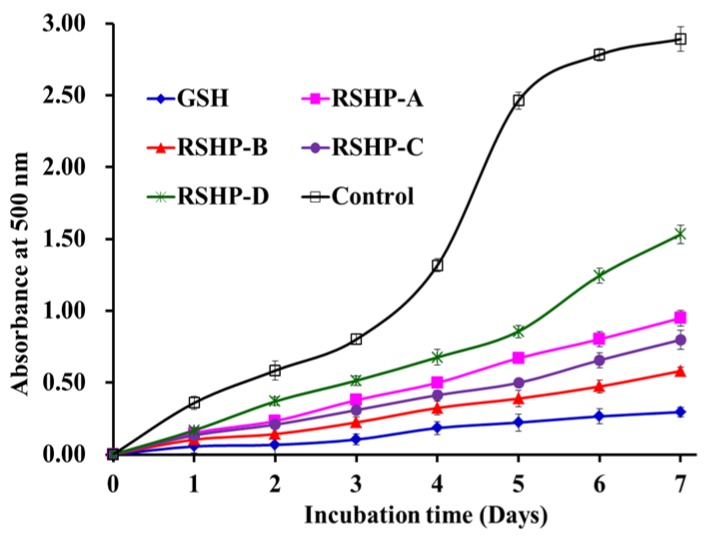
Lipid peroxidation inhibition abilities of the four isolated APs (RSHP-A~D) from protein hydrolysate of red stingray (*D. akajei*) cartilages. All data are presented as mean ± SD (*n* = 3).

Lipid peroxidation is a complex process that involves the formation and propagation of lipid radicals and lipid hydroperoxides in the presence of oxygen [2]. Each assay, such as DPPH, hydroxyl, superoxide anion, and ABTS cation radical scavenging assays measured an antioxidant property that represents a different mechanism, which could not reflect the multiple mechanisms, by which the sample acted as an antioxidant to inhibit lipid oxidation in a food system and living organisms [51]. Therefore, the activities of RSHP-A, RSHP-B, RSHP-C, and RSHP-D against the peroxidation of linoleic acid were investigated and compared to that of the positive control (GSH) and negative control (without antioxidant) in this section (Figure 9). The inhibiting ability of RSHP-B was higher than those of RSHP-A, RSHP-C, and RSHP-D. However, the inhibiting ability of four isolated APs (RSHP-A~D) was lower than that of the positive control of GSH. The result of lipid peroxidation inhibition assay suggested that RSHP-B might inhibit the lipid peroxidation propagation cycle through reacting with peroxyl radicals. In addition, the activity of RSHP-B on lipid peroxidation inhibition slightly less than GSH can be compensable by increasing the dose.

## 3. Discussion

The structural characteristics of APs provide guides for the evaluation of food-derived proteins as potential precursors of APs and predict the possible release of APs from various proteins while using appropriate proteases [1].

Acidic and basic amino acid residues play critical roles in the metal ion chelating activity, which is related to the carboxyl and amino groups in their side chains [56]. The carboxyl residues of acidic amino acids are deprotonated for rendering the complex formation with metal ions, and the amino nitrogen loses its proton nitrogen to allow for unshared pairs of electrons of nitrogen to bind with metal ions [53]. Memarpoor-Yazdi et al. reported similar results, who found that the basic (Arg) and acidic (Asp and Glu) amino acid residues in the sequences of NTDGSTDYGILQINSR and LDEPDPLI were critical to their antioxidant activities [57]. Díaz et al. found that Glu residue is an effective cation chelator that forms complexes with calcium and zinc ions and it may contribute to the antioxidant activity [58]. Therefore, the Glu residue in RSHP-B, RSHP-C and RSHP-D, and Arg residue in RSHP-A might be favorable for their antioxidant activities.

Mirzaei et al. reported that the pyrrolidine ring of Pro residue can interact with the secondary structure of the peptide, thereby increasing the flexibility, and it is also capable of quenching singlet oxygen due to its low ionization potential [59]. Samaranayaka and Li-Chan reported that Pro residue played an important role in the activity of AP that was purified from *Saccharomyces cerevisiae* protein hydrolysate [60]. Therefore, the Pro residue in the amino acid sequences of RSHP-A and RSHP-B should enhance their radical-scavenging activities.

Aromatic amino acid residues, such as Phe, Tyr, His, and Trp, and hydrophobic amino acid residues, including Ala, Val, and Leu, have been reported as critical to the activities of APs [1]. The results from Guo et al. indicated that hydrophobic amino acid residues (e.g., Val, Ile, and Leu) and aromatic amino acid residues (Phe, His, Tyr, and Trp) can enhance the radical-scavenging abilities of APs from Chinese cherry seeds [61]. Therefore, the presence of aromatic and hydrophobic amino acid residues in RSHP-A (Val), RSHP-B (Ile and His), RSHP-C (Leu), and RSHP-D (Ile) should have a positive impact on their radical-scavenging and lipid peroxidation inhibitory activities.

In addition, the activities of APs are dependent on their molecular size. Short peptides with 2–10 amino acid residues have stronger radical-scavenging and lipid peroxidation inhibition activities than their parent native proteins or long-chain peptides [1]. RSHP-A~D exhibited good antioxidant activities, which suggested that they could more effectively and easily interact with free radicals and inhibit the propagation cycles of lipid peroxidation [33]. However, RSHP-B showed the strongest radical scavenging activity (DPPH•, HO•, and ABTS^+^•), reducing power, and lipid peroxidation inhibition activities, RSHP-A showed the strongest $O_{2}^{-}$• scavenging activity and RSHP-C showed the highest Fe^2+^-chelating ability among the four isolated APs. There was no consistent trend in the different tested assays. Therefore, more detailed study should be performed to clarify the relationship between the activity and structure of the four isolated APs (RSHP-A~D).

## 4. Experimental Section

### 4.1. Materials

Red stingray (*D. akajei*) was purchased from Nanzhen Market in Zhoushan city of China and its cartilages were manually removed with a filleting knife, washed with cold DW, cut into small pieces (about 0.5 cm^2^), and then stored at −20 °C. Bovine serum albumin (BSA), DEAE-52 cellulose, D101 macroporous resin, and Sephadex G-15 were purchased from Shanghai Source Poly Biological Technology Co., Ltd. (Shanghai, China). CAN of LC grade and trifluoroacetic acid (TFA) were purchased from Thermo Fisher Scientific Co., Ltd. (Shanghai, China). Four APs (VPR, IEPH, LEEEE, and IEEEQ) with a purity higher than 98% were synthesized in Shanghai Apeptide Co., Ltd. (Shanghai, China).

### 4.2. Preparation of Water-Soluble Proteins and Hydrolysate from Red Stingray Cartilages

The preparation of water-soluble proteins: The small pieces of cartilages (about 0.5 cm^2^) were broken using a hammer, minced to homogenate, and then soaked in 1.0 M guanidine hydrochloride with a solid to solvent ratio of 1:5 (*w*/*v*) for 48 h, and the liquid supernatant was collected by centrifugation at 4 °C, 12,000× *g* for 10 min and then dialyzed (MW 1 kDa) against 25 volumes of DW for 12 h with solution change every 4 h. Finally, the resulted dialysate (water-soluble proteins) was collected and freeze-dried.

Enzymatic hydrolysis of water-soluble proteins: The freeze-dried water-soluble proteins was dissolved (5 % *w*/*v*) in 0.2 M phosphate buffer solution (PBS, pH 8.0) and hydrolyzed while using trypsin with a total enzyme dose of 2.5% at 40 °C for 4 h. The process was terminated by heating the hydrolysate to 95 °C for 10 min. Finally, the hydrolysate was centrifuged at 9000× *g* for 15 min, and the supernatant (RSH) was desalted using D101 macroporous resin, freeze-dried, and stored at −20 °C. The concentrations of RSH and its fractions were expressed as mg protein/mL and determined by the dye binding method of Bradford (1976), with BSA as the standard protein

### 4.3. Isolation of Peptides from RSH

RSH was fractionated into three fractions, termed as RSH-I (MW < 3 kDa), RSH-II (3 < MW < 10 kDa), and RSH-III (MW > 10 kDa), by 3 and 10 kDa ultrafiltration membranes in the Labscale TFF System of Millipor Ltd. (Billerica, MA, USA).

The RSH-I solution (5 mL, 40.0 mg protein/mL) was injected into a DEAE-52 cellulose column (1.6 × 80 cm) pre-equilibrated with DW, and then stepwise eluted with 150 mL DW, 0.1 M, 0.5 M, and 1.0 M NaCl solution at a flow rate of 1.0 mL/min, respectively. Each eluted fraction (5 mL) was collected and measured at 280 nm. Finally, four fractions (RSH-I-1 to RSH-I-4) were pooled on their chromatographic peaks and lyophilized.

RSH-I-4 solution (5 mL, 10.0 mg protein/mL) was fractionated on a Sephadex G-15 column (2.6 × 160 cm) eluted with DW at a flow rate of 0.6 mL/min. Each elate (3 mL) was monitored at 280 nm and then collected on the chromatographic peaks, and two fractions (Frac.1 and Frac.2) were prepared and lyophilized.

A Thermo C-18 column (4.6 × 250 mm, 5μm) (Thermo Co., Ltd., Yokohama, Japan) attached to an Agilent 1260 HPLC system (Agilent Ltd., Santa Rosa, CA, USA) further purified Frac.1. The sample (20 µL) was injected into the HPLC column, eluted with a linear gradient of ACN (0–40% in 0–25 min) in 0.1% TFA at a flow rate of 0.8 mL/min, and monitored at 280 nm. Finally, four APs (RSHP-A~D) were isolated and lyophilized.

### 4.4. Amino Acid Sequence and Molecular Mass Analysis

Amino acid sequences and molecular masses of RSHP-A, RSHP-B, RSHP-C, and RSHP-D were measured on the previous method [51]. The amino acid sequences of RSHP-A, RSHP-B, RSHP-C, and RSHP-D were determined on an Applied Biosystems 494 protein sequencer (Perkin Elmer/Applied Biosystems Inc., Foster City, CA, USA). Edman degradation was performed according to the standard program that was supplied by Applied Biosystems.

Molecular masses of RSHP-A, RSHP-B, RSHP-C, and RSHP-D were determined using a Q-TOF mass spectrometer (Micromass, Waters, Milford, MA, USA), coupled with an electrospray ionization (ESI) source. Ionization was carried out in a positive mode with a capillary voltage of 3500 V. Nitrogen was maintained at 40 psi for nebulization and 9 L/min at 350 °C for the evaporation temperature. Data were collected in centroid mode from *m/z* 100 to 2000.

### 4.5. Antioxidant Activity

#### 4.5.1. Radical Scavenging Activity

The radical scavenging and lipid peroxidation inhibition assays were measured on the previous method [38], and the half elimination ratio (EC_50_) was defined as the concentration where a sample caused a 50% decrease of the initial concentration of DPPH•, HO•, $O_{2}^{-}$•, and ABTS^+^•, respectively.

#### 4.5.2. Fe^2+^-Chelating Assay

The Fe^2+^-chelating assay was performed in accordance with the previous report [62,63]. In brief, the mixed reaction solution (1815 μL DW, 450 μL sample (0, 0.25, 0.5, 0.75, and 1.0 mg/mL) and 45 μL FeSO_4_ (2 mM)) was energetically shaken and maintained for 0.5 h. After that, 90 μL of ferrozine (5 mM) were added into reaction solution, mixed well, and determined the absorbance at 562 nm. DW was given as the blank control. The Fe^2+^-chelating ability was calculated using the following formula: Fe^2+^-chelating ability (%) = [(Absorbance of blank control − Absorbance of sample)/Absorbance of blank control] × 100.

#### 4.5.3. Reducing Power Assay

The reducing power assay was carried out following the previous method [64]. 2.5 mL of 1% K_3_Fe(CN)_6_ solution was blend with 2.0 mL of peptide solution and incubated at 50 °C for 0.5 h. After that, 1.5 mL of 10% trichloroacetic acid was added into the mixed solution. Finally, 2.0 mL of the upper layer, 0.5 mL of 0.1% aqueous FeCl_3_, and 2.0 mL of DW were mixed, and the absorbance at 700 nm was applied to record the reaction mixture.

#### 4.5.4. Lipid Peroxidation Inhibition Assay

The lipid peroxidation inhibition capacities of APs were measured in a linoleic acid model system, according to the previous methods [65,66]. Briefly, a sample (5.0 mg) was dissolved in 10 mL of PBS (50 mM, pH 7.0) and added to 0.13 mL of a linoleic acid solution and 10 mL of 99.5% ethanol. Subsequently, the total volume was adjusted to 25 mL with DW. The mixture was incubated in a conical flask with a screw cap at 40 °C in a dark room, and the degree of oxidation was evaluated by measuring the Fe(SCN)_3_ values. The reaction solution (100 μL), incubated in the linoleic acid model system, was mixed with 4.7 mL of 75% ethanol, 0.1 mL of 30% NH_4_SCN, and 0.1 mL of 20 mM FeCl_2_ solution in 3.5% HCl. After 3 min, the thiocyanate value was measured at 500 nm following color development with FeCl_2_ and thiocyanate at different intervals during the incubation period at 40 °C.

### 4.6. Statistical Analysis

All data are expressed as the mean ± standard deviation (SD, *n* = 3). An ANOVA test using SPSS 19.0 (Statistical Program for Social Sciences, SPSS Corporation, Chicago, IL, USA) was used to analyze the experimental data. A *p*-value of less than 0.05 was considered to be statistically significant.

## 5. Conclusions

In the experiment, four APs (RSHP-A~D) were isolated from water-soluble protein hydrolysate of red stingray (*D. akajei*) cartilages using ultrafiltration and chromatographic methods, and their amino acid sequences were identified as VPR, IEPH, LEEEE, and IEEEQ, respectively. Among the four isolated APs, IEPH showed the strongest scavenging activity on DPPH•, HO•, and ABTS^+^•, reducing power, and lipid peroxidation inhibition activities; VPR showed the strongest $O_{2}^{-}$• scavenging activity; and, LEEEE showed the highest Fe^2+^-chelating ability. Subsequently, APs (RSHP-A~D) from water-soluble protein hydrolysate of red stingray cartilages may be possible to serve as antioxidant candidates for new functional foods. Further studies will be done to purify and identify more APs from Frac.1, and more detailed studies on the safety, stability, and structure-activity relationship of the purified APs will also be needed.

## Figures and Tables

**Table 1:** Retention time (min), amino acid sequences, molecular masses, and radical scavenging activities of four antioxidant peptides (APs) (RSHP-A~D) from water-soluble protein hydrolysate of red stingray (*D. akajei*) cartilages.

		RSHP-A	RSHP-B	RSHP-C	RSHP-D
Retention time (min)		9.434	13.435	14.137	18.610
Amino acid sequence		VPR	IEPH	LEEEE	IEEEQ
Theoretical mass/ observed mass (Da)		370.44/ 370.46	494.52/ 494.55	647.69/ 647.64	646.62/ 646.66
EC_50_ (mg/mL)	DPPH•	4.61	0.77	0.08	0.15
	HO•	1.90	0.46	0.17	0.11
	$O_{2}^{-}$•	3.69	0.70	0.15	0.19
	ABTS^+^•	4.01	1.30	0.16	0.18
